# Shaping the lipid composition of bacterial membranes for membrane protein production

**DOI:** 10.1186/s12934-019-1182-1

**Published:** 2019-08-10

**Authors:** Kerstin Kanonenberg, Jorge Royes, Alexej Kedrov, Gereon Poschmann, Federica Angius, Audrey Solgadi, Olivia Spitz, Diana Kleinschrodt, Kai Stühler, Bruno Miroux, Lutz Schmitt

**Affiliations:** 1 Institute of Biochemistry, Heinrich-Heine-University Duesseldorf, Universitaetsstr. 1, 40225 Duesseldorf, Germany; 2 Present Address: CNRS, UMR5086 “Molecular Microbiology and Structural Biochemistry”, Université de Lyon, 7 Passage du vercors, 69007 Lyon, France; 3 Laboratoire de Biologie Physico-Chimique des Protéines Membranaires, UMR7099, CNRS, IBPC, Université Paris Diderot, Sorbonne Paris Cité, 13 rue Pierre et Marie Curie, 75005 Paris, France; 4 Molecular Proteomics Laboratory, Biologisch Medizinisches Forschungszentrum (BMFZ), Heinrich-Heine-University Duesseldorf, Duesseldorf, Germany; 5 Present Address: Department of Microbiology, Institute for Water and Wetland Research, Heyendaalseweg 135, 6525 Nijmegen, The Netherlands; 6 Institut Paris Saclay d’Innovation Thérapeutique, INSERM, CNRS, - Plateforme SAMM, Université Paris-Saclay, Châtenay-Malabry, France

**Keywords:** Strain engineering, Membrane engineering, Solubilization, Lipidome, Membrane protein

## Abstract

**Background:**

The overexpression and purification of membrane proteins is a bottleneck in biotechnology and structural biology. *E. coli* remains the host of choice for membrane protein production. To date, most of the efforts have focused on genetically tuning of expression systems and shaping membrane composition to improve membrane protein production remained largely unexplored.

**Results:**

In *E. coli* C41(DE3) strain, we deleted two transporters involved in fatty acid metabolism (OmpF and AcrB), which are also recalcitrant contaminants crystallizing even at low concentration. Engineered expression hosts presented an enhanced fitness and improved folding of target membrane proteins, which correlated with an altered membrane fluidity. We demonstrated the scope of this approach by overproducing several membrane proteins (4 different ABC transporters, YidC and SecYEG).

**Conclusions:**

In summary*, E. coli* membrane engineering unprecedentedly increases the quality and yield of membrane protein preparations. This strategy opens a new field for membrane protein production, complementary to gene expression tuning.

**Electronic supplementary material:**

The online version of this article (10.1186/s12934-019-1182-1) contains supplementary material, which is available to authorized users.

## Introduction

All sequenced genomes contain about 20–30% of genes encoding membrane proteins (MP) [1]. However, they are still underrepresented in biochemical and structural studies, despite their undeniable physiological and medical importance—about 70% of all drug targets are membrane proteins. The bottleneck of developing drugs targeting membrane proteins is the overproduction and the requirement for pure, homogeneous, and folded protein(s). *Escherichia coli* (*E. coli*) remains first choice for membrane protein production and contributed to 470 unique membrane protein structures (UMPS, 41 from eukaryotic origin and 248 from bacteria other than *E. coli*) over 722 UMPS deposited in the protein data bank in April 2018 [2]. Despite this, the difficulties frequently encountered upon overproduction of MP in *E. coli* are: (i) the toxicity of an excess of target MP mRNA levels, (ii) the overloading of the translation and secretion machineries [3, 4], (iii) the toxicity of the overproduced MP [5], and (iv) the lipid composition of the microbial host. So far, optimization of membrane protein production has been achieved almost exclusively by tuning transcription of the target gene [4, 6]. In the arabinose expression system, a correlation has been observed between the amount of inducer and the formation of inclusion bodies (IB) of the recombinant MP [7]. In the T7 RNA polymerase based expression system, tuning of the promoter has been achieved by genetic selection of bacterial mutants. For instance, the C41(DE3) strain has been isolated from BL21(DE3), and C43(DE3) was further selected based on the toxicity of AtpF protein in C41(DE3) [8]. A subtle change in the AtpF transcriptional time course of expression in C43(DE3) was sufficient to restore the fitness of the cell, to avoid IB formation and to induce internal membrane proliferation [9]. Other mutations were identified in the T7RNA polymerase gene [10, 11]: The human sulfide quinone reductase, which formed IB in all previously tested strains, could be targeted and folded into membranes in a recently isolated mutant strain C45(DE3) [10]. However, tuning the promoter is sometimes not sufficient. Instead, a new strategy has emerged, mostly in unicellular eukaryotic expression systems, which focuses on engineering the lipid composition of the membrane [12]. In this study, our aim was to modulate *E. coli* membrane composition to accommodate large amounts of MP. The outer membrane pores OmpF and FadL have been shown to impact the fatty acid composition of the phospholipids and the membrane integrity [13]. In contrast, the inner membrane tripartite efflux pump AcrAB-TolC is involved in the efflux of many molecules, including lipids [14]. We therefore postulated that the deletion of both, OmpF and AcrAB, should be advantageous for two reasons. First, it should modify the membrane composition and its tolerance to MP overproduction; and second, we expected an improvement in the purity of the recombinant MP. Indeed, OmpF and AcrB, are the principal contaminants when MP are purified using IMAC affinity chromatography [15]. Both proteins crystallize easily even from very low concentrations [16–18], which is a major issue in structural biology of MP. To test our hypothesis, we constructed a derivative of *E. coli* C41(DE3) strain lacking *acrAB* and *ompF* genes. To the best of our knowledge, the only study using a deletion of four outer membrane proteins, *ompA*, *ompC*, *ompF* and *lanB*, was used for the expression of outer membrane proteins [19, 20]. For structural studies, two deletions of *acrAB* have been studied [21, 22]. However, an *ompF* and/or *ompF*-*acrAB* double deletion has not been investigated. The deletion of these two genes did not only increase the amount of overproduced membrane proteins, but also enhanced their folding as reflected by an increased solubilization efficiency with mild surfactants. Membrane lipid composition analysis provided a rational explanation of the improved extraction and purification yield of the target membrane proteins.

## Results

### Construction of *E. coli* C41(DE3)∆(*ompF*-*acrAB*)

Subsequent to the deletion of *ompF, acrAB* was deleted by employing the lambda-red recombinase system. Both genomic deletions were confirmed by PCR and subsequent sequencing of the full genome of C41(DE3)∆(*ompF*-*acrAB*). The comparison to the genome of the parental strain C41(DE3) (GenBank ID: NZ_CP010585.1) [23] eliminated the possibility of other modifications apart from the desired deletions.

**Fig. 1:**
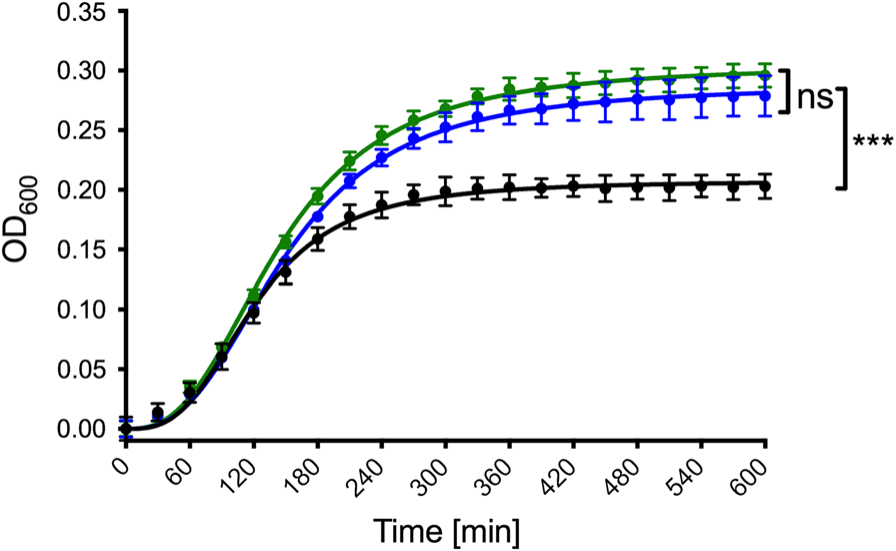
Net growth curves of *E. coli* C41(DE3) (black line), *E. coli* C41(DE3)∆(*ompF*) (blue line) and *E. coli* C41(DE3)∆(*omp*F-*acrAB*) (green line). The final ODs were analyzed using a one-way ANOVA followed by a Tukey multiple comparisons test. Error bars represent SD of two individual measurements. ns: not significant, ***p < 0.001

We compared the growth of the double deletion strain, C41(DE3)∆(*ompF*-*acrAB*), to C41(DE3) and the single deletion strain C41(DE3)*∆(ompF)*. Our results demonstrate that under the tested condition the deletion of *ompF* from the genome results in growth to approximately 50% higher ODs, while the additional deletion of *acrAB* does not further influence the growth behavior (Fig. 1). This change in growth behavior is further reflected by an increase of the growth rate and a decrease of the generation time of the deletion strains (Table 1).

**Table 1:** Growth rates (µ) and generation times (t_d_) of *E. coli* C41(DE3), *E. coli* C41(DE3)∆(*ompF*) and *E. coli* C41(DE3)∆(*omp*F-*acrAB*) calculated from the exponential phases of the growth curves

Strain	Growth rate µ [min^−1^]	Generation time t_d_ [min]
C41(DE3)	0.026 ± 0.004	26.92 ± 4.18
C41(DE3)∆(*ompF*)	0.028 ± 0.003	24.73 ± 2.65
C41(DE3)∆(*omp*F-*acrAB*)	0.033 ± 0.003	21.28 ± 1.96

### Overexpression of ABC transporters in *E. coli* C41(DE3), C41(DE3)∆(*ompF*) and C41(DE3)∆(*ompF*-*acrAB*)

**Fig. 2:**
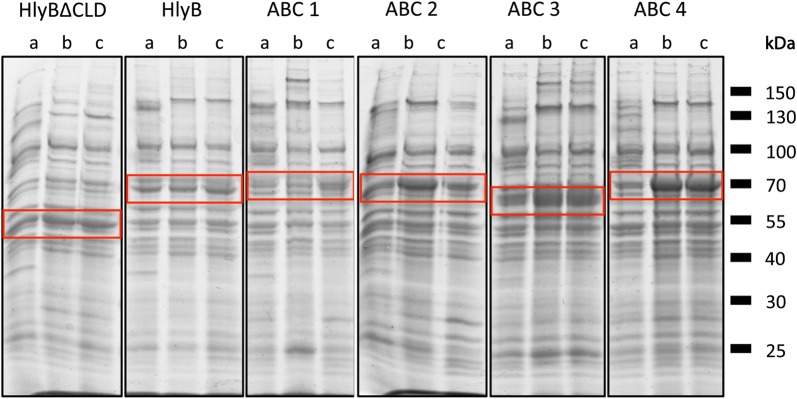
Overexpression of membrane proteins in *E. coli* C41(DE3) (a), C41(DE3)∆(*ompF*) (b) and C41(DE3)∆(*ompF*-*acrAB*) (c). Equal amounts (50 µg) of isolated membranes were loaded on 10% SDS-PAGE and stained with Coomassie Brilliant Blue. Target proteins are marked by red boxes. Independent expressions were performed three times with similar results

Our aim was not to perform a precise quantification of protein production but rather to check that recombinant MP production was not impaired in our mutants. We tested overexpression of ABC transporters, a broad and vital class of membrane proteins. In total, six prokaryotic ABC transporters both homologous (HlyB∆CLD or HlyB) and heterologous (termed ABC1 to ABC4 in this study), were analyzed. The total amounts of membrane-inserted target proteins were compared by SDS-PAGE. Equal amounts of total protein were loaded on the gels (Fig. 2).

For five ABC transporters, i.e. HlyB∆CLD, HlyB, ABC2, ABC3, and ABC4, production levels appeared improve by using the OmpF-depleted strain. In those cases, the additional deletion of *acrAB* either had no further enhancement effect or slightly reduced the expression levels (ABC2). However, one ABC transporter (ABC1) seemed overproduced in considerable amounts only in C41(DE3)∆(*ompF*-*acrAB*) (Fig. 2).

### High yield purification of HlyB in mild detergent from C41(DE3)∆(*ompF*-*acrAB*) membranes

**Fig. 3:**
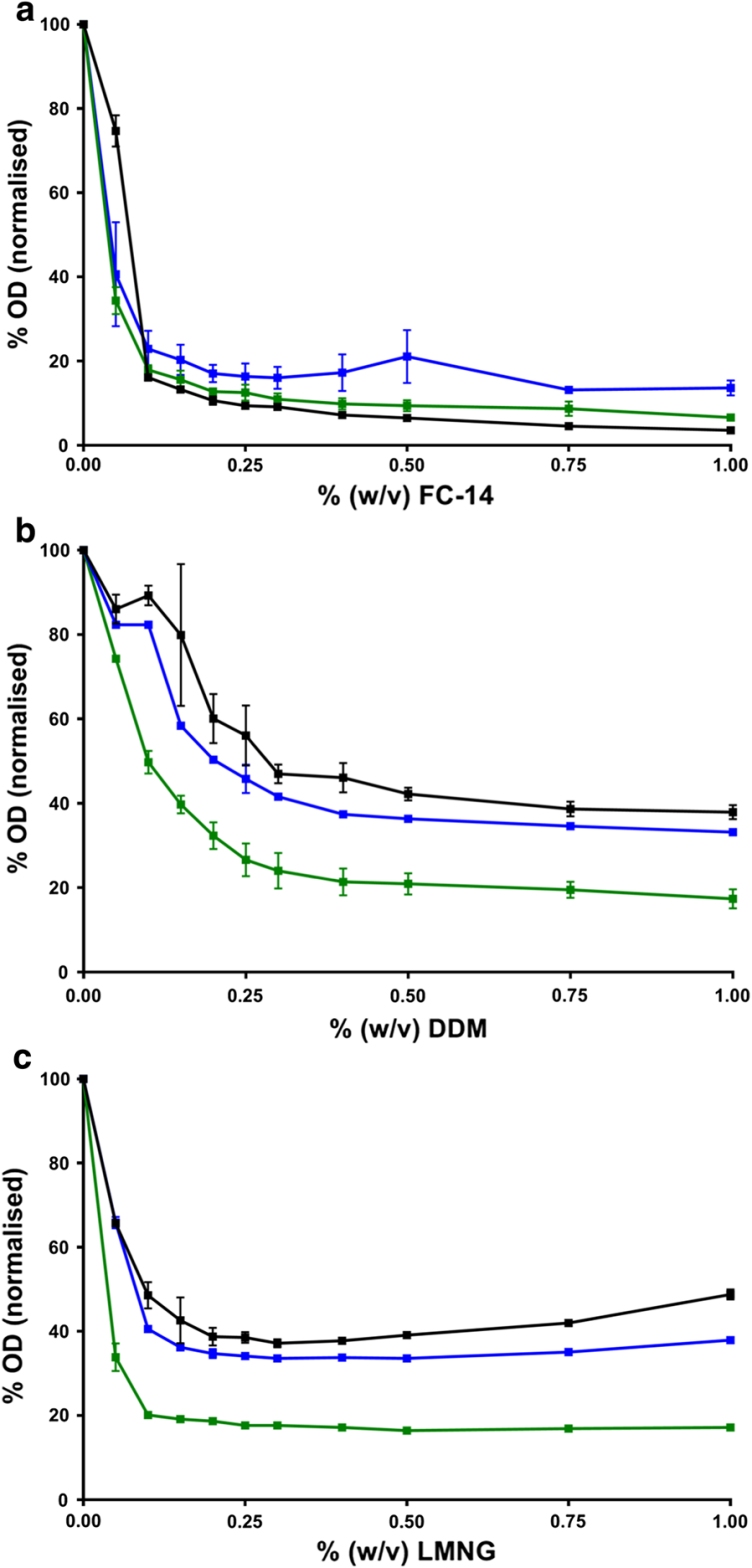
Visualization of the solubilization efficiency of the three detergents **a** FC-14, **b** DDM and **c** LMNG on HlyB-containing membranes from *E. coli* C41(DE3) (black line), C41(DE3)∆(*ompF*) (blue line), and C41(DE3)∆(*ompF*-*acrAB*) (green line). Measurements were repeated in triplicates, error bars represent SD

**Fig. 4:**
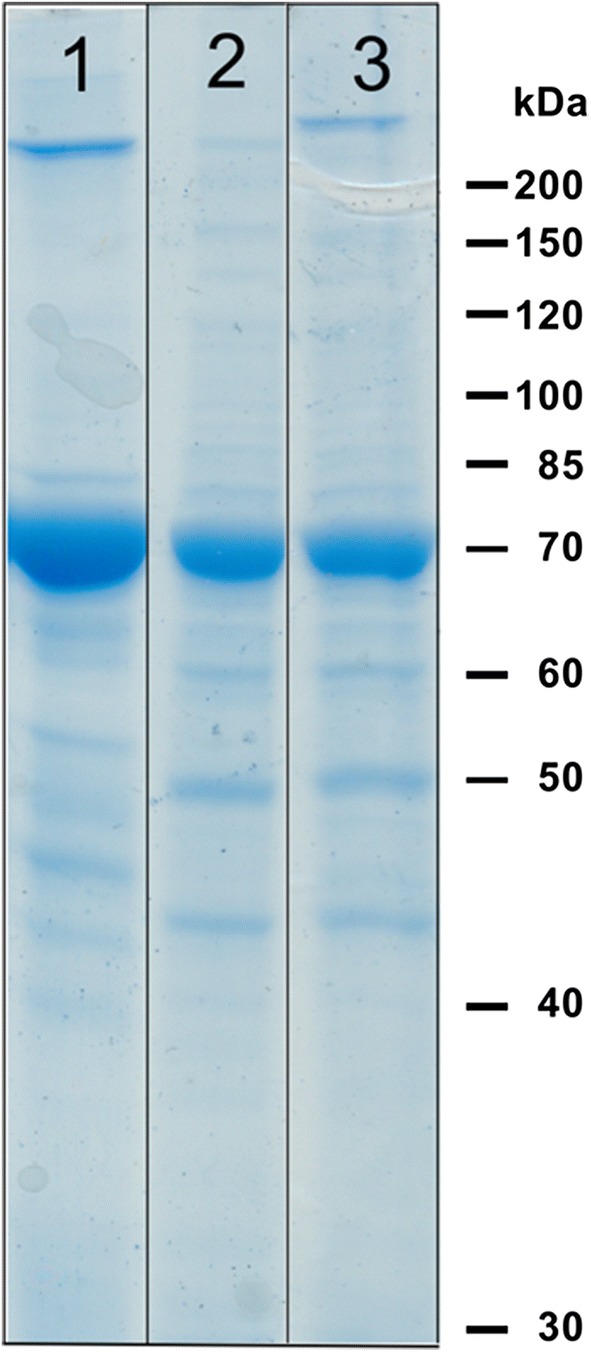
Coomassie Brilliant Blue stained 10% SDS-PAGE of the purification of HlyB from *E. coli* C41(DE3)∆(*ompF*-*acrAB*) employing different detergent-combinations for solubilization (first detergent) and subsequent IMAC (second detergent). (1) Fos-choline 14/DDM, (2) DDM/DDM, (3) TX-100/DDM. Shown are the pooled eluate fractions of equal volume. Independent expressions and purifications were performed two times with similar results

HlyB has been shown previously to be exclusively purified using Fos-Choline (FC)-derived detergents [24]. We tested several detergents by determining the turbidity of membrane samples after addition of detergent at increasing concentrations. When using the zwitterionic detergent FC-14 the solubilization efficiencies of all membranes were similar. However, when employing the non-ionic detergents DDM or LMNG, solubilization of HlyB was increased by approximately 20% from C41(DE3)∆(*ompF*-*acrAB*) membranes (Fig. 3). Next, HlyB was purified from C41(DE3)∆(*ompF*-*acrAB*) membranes utilizing DDM and TX-100. In contrast to previous studies [24], the purification with non-ionic detergents resulted in approximately 80% yield of HlyB with comparable purity, confirming the improved extraction of HlyB from the membrane (Fig. 4).

### Purification of SecYEG and YidC from *E. coli* C41(DE3), C41(DE3)∆(*ompF*), and C41(DE3)∆(*ompF*-*acrAB*)

**Fig. 5:**
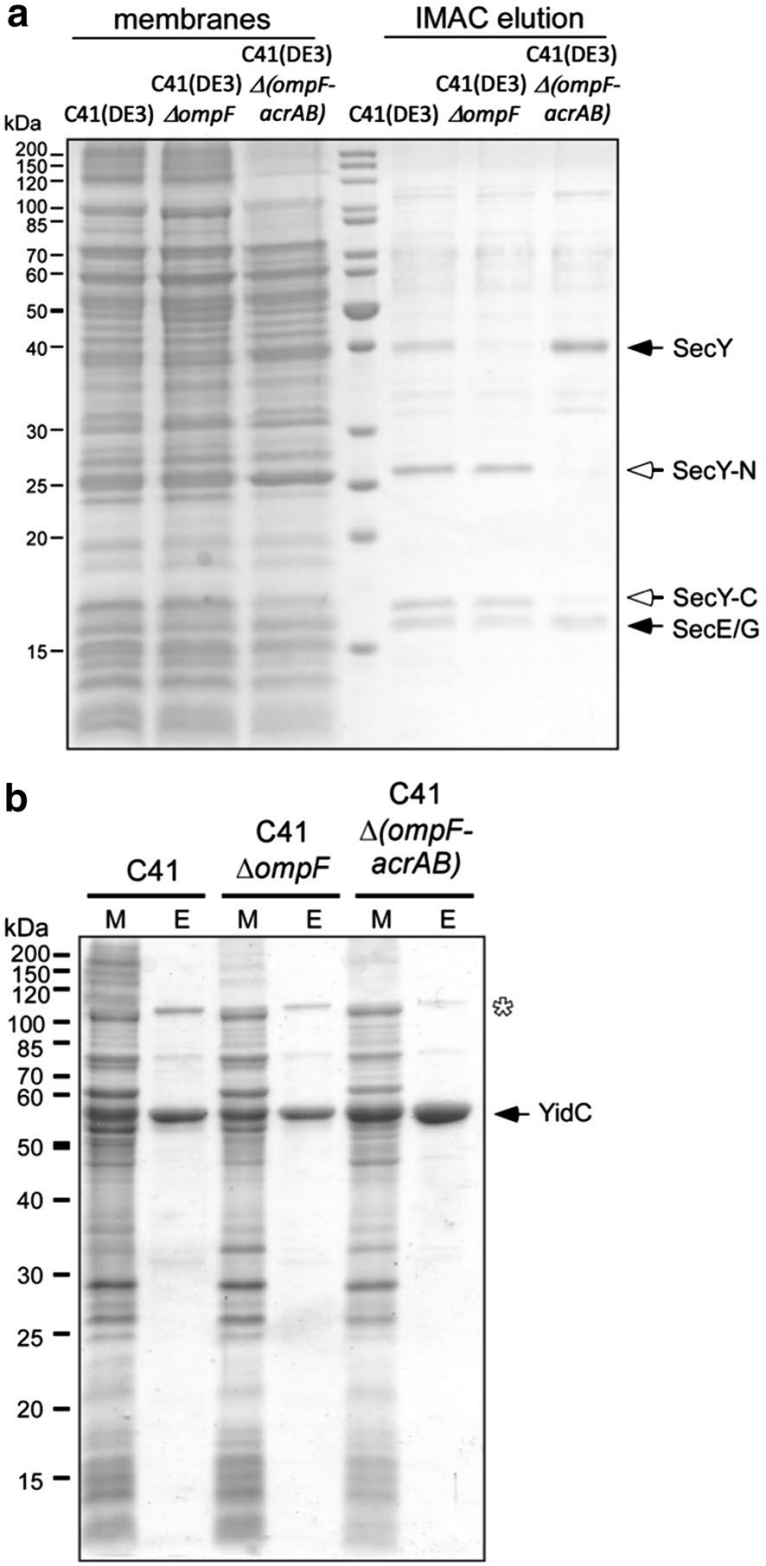
Purification of SecYEG (**a**) and YidC (**b**) from *E. coli* C41(DE3), C41(DE3)∆(*ompF*) and C41(DE3)∆(*ompF*-*acrAB*). M: starting material, E: eluted protein. Independent purifications were performed three times for SecYEG and two times for YidC with similar results

The translocon SecYEG and the insertase YidC are well-known and extensively studied *E. coli* membrane proteins, thus rendering them ideal candidates to perform test purifications from C41(DE3), C41(DE3)∆(*ompF*) and C41(DE3)∆(*ompF*-*acrAB*) strains. For those, equal amounts of membranes were used and overall yield and purity were analyzed by SDS-PAGE. The translocon subunit SecY was found to be partially and nearly completely degraded in the membranes of C41(DE3) and C41(DE3)*∆*(*ompF*), respectively, which resulted in the purification of fragments termed SecY-N and SecY-C (Fig. 5). This degradation was not affected by adding protease inhibitors to the membranes prior to purification, which indicated a proteolytic digest during cellular protein overexpression. However, C41(DE3)∆(*ompF*-*acrAB*) strongly reduced the degradation of SecY, which resulted in a clear band for the full-length protein (Fig. 5). The other components of the translocon SecE and SecG, were unaffected by the choice of expression strain.

For YidC, the effects of using C41(DE3)∆(*ompF*-*acrAB*) for the overexpression were less pronounced; however, a substantial increase in the yield of purified protein was observed, probably resulting from an increase in expression levels. This is reflected by an approximately twofold more intense band in the starting material (Fig. 5). We additionally analyzed the impurity at ~ 100 kDa, which appeared the most intense in C41(DE3) and C41(DE3)∆(*ompF*)-based isolates. Mass spectrometric analysis revealed that the band was largely composed of 2-oxoglutarate dehydrogenase and AcrB, thus explaining the impurity depletion in C41(DE3)∆ (*ompF*-*acrAB*) strain.

### Analysis of the membrane density by density gradient centrifugation

**Fig. 6:**
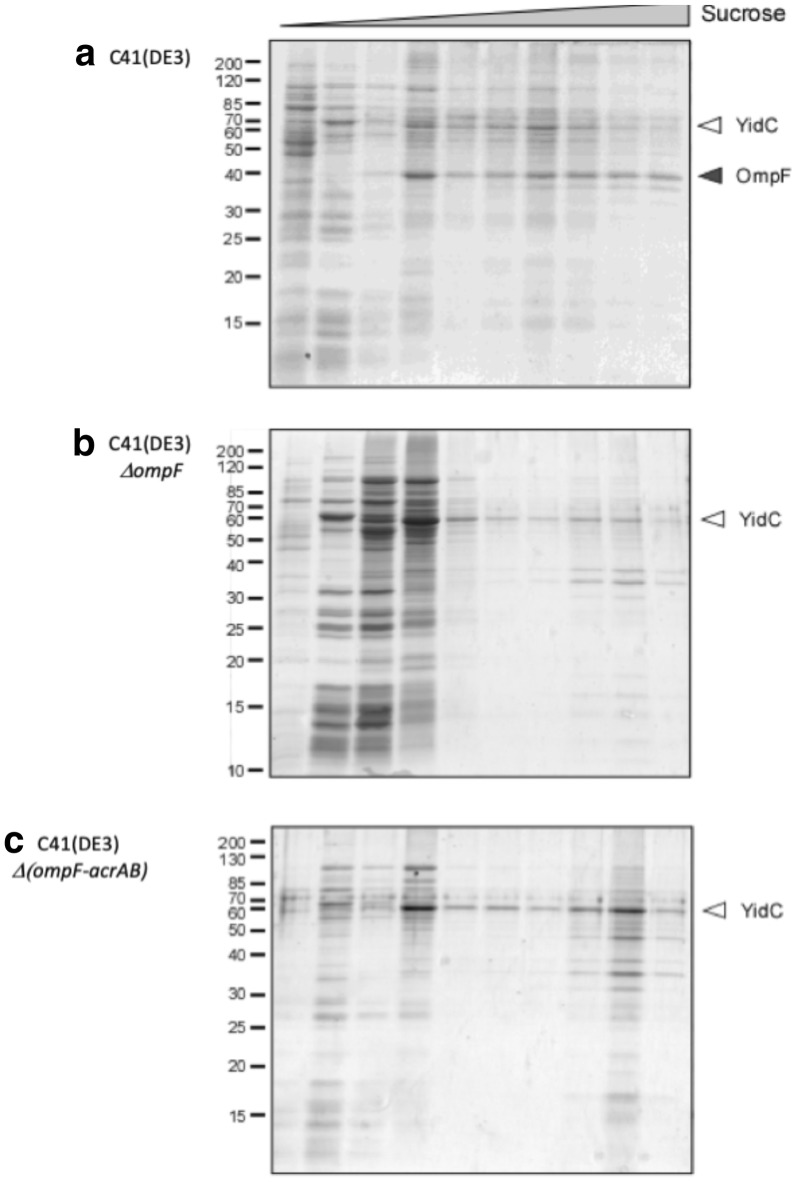
Results of the density-gradient centrifugation of membranes purified from YidC-expressing strains **a** C41(DE3), **b** C41(DE3)∆(*ompF*) and **c** C41(DE3)∆(*ompF*-*acrAB*). Sucrose concentration ranged from 20 to 70%. YidC can be recognized as a prominent band at 60 kDa. Independent purifications and sucrose-gradient centrifugations were performed two times with similar results

YidC-containing membranes were fractionated and further analyzed by centrifugation in a sucrose gradient (20–70%) and subsequent SDS-PAGE of collected fractions (Fig. 6). For all strains early fractions (20–30% sucrose) contained a large number of proteins and exhibited the characteristic pattern of ribosomal proteins ranging between 15 and 35 kDa, likely containing proteins loosely attached to membranes. Fractions of C41(DE3) also show a prominent band at approximately 40 kDa. Due to its absence from the other strains, this band very likely corresponds to OmpF. The distribution of YidC through the gradient differed between the three examined strains. In the parental strain, YidC was most prominent in fractions containing 30–50% sucrose. The deletion of *ompF* gene resulted in a local concentration of YidC in fractions containing approximately 30% sucrose, but also in later fractions (50–70% sucrose). The additional deletion of a*crAB* resulted in an even higher accumulation of YidC in these late fractions (Fig. 6). Our results suggest an alteration of the density of the membranes, which was further analyzed by mass spectrometric measurements of the lipidome.

### Mass spectrometry analysis of phospholipids

**Fig. 7:**
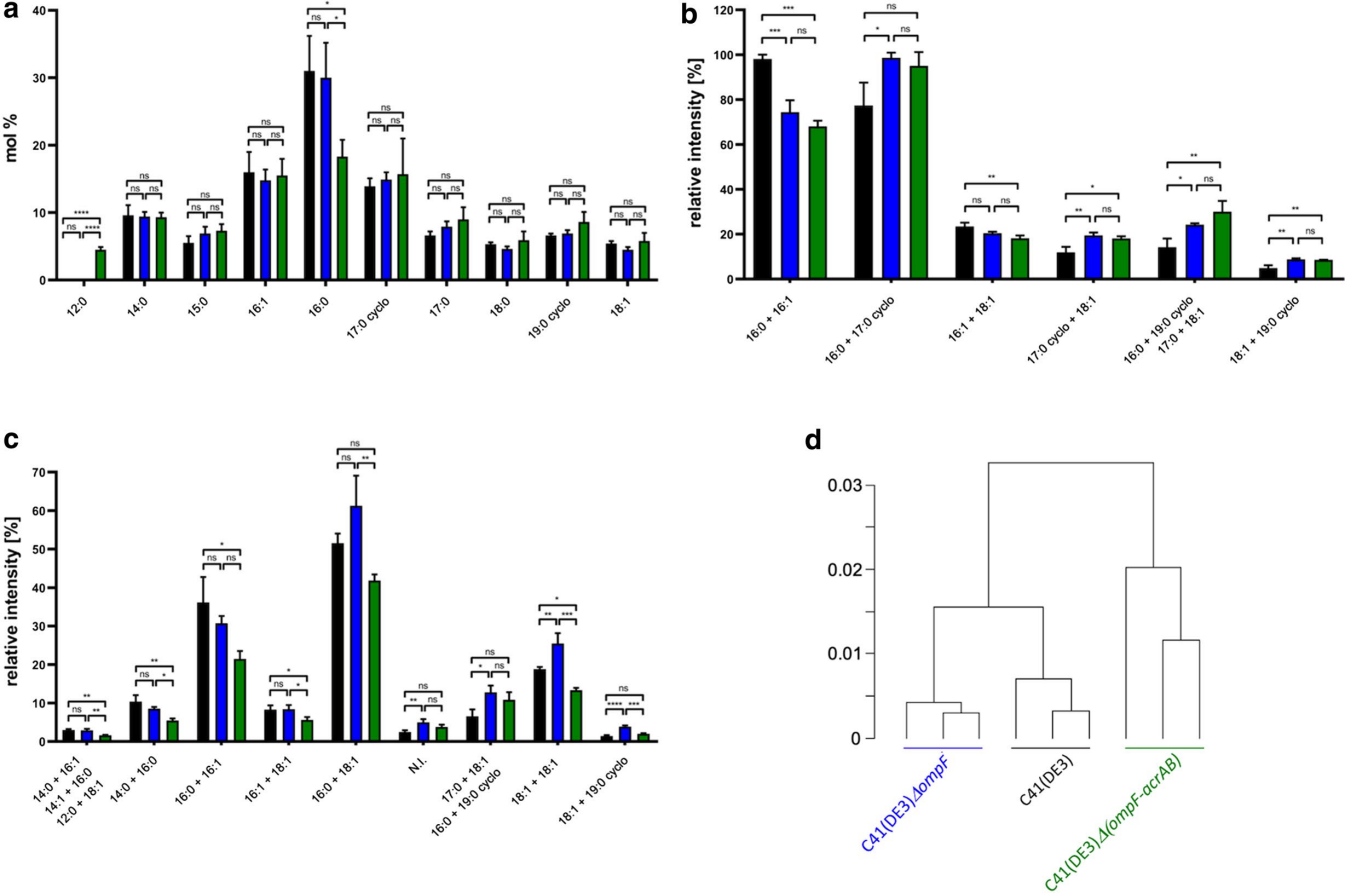
**a** Phospholipid FA analysis, **b** PE speciation analysis, **c** PG speciation analysis and **d** hierarchical cluster visualizing PCA results for C41(DE3) (black), C41(DE3)∆(*ompF*) (blue), and C41(DE3)∆(*ompF*-*acrAB*) (green) overexpressing HlyB. P-values were calculated using a one-way ANOVA followed by a Tukey multiple comparisons test. ns: not significant (p > 0.05), *p ≤ 0.05, **p ≤ 0.01, ***p ≤ 0.001, ****p ≤ 0.0001. Error bars represent SD

**Fig. 8:**
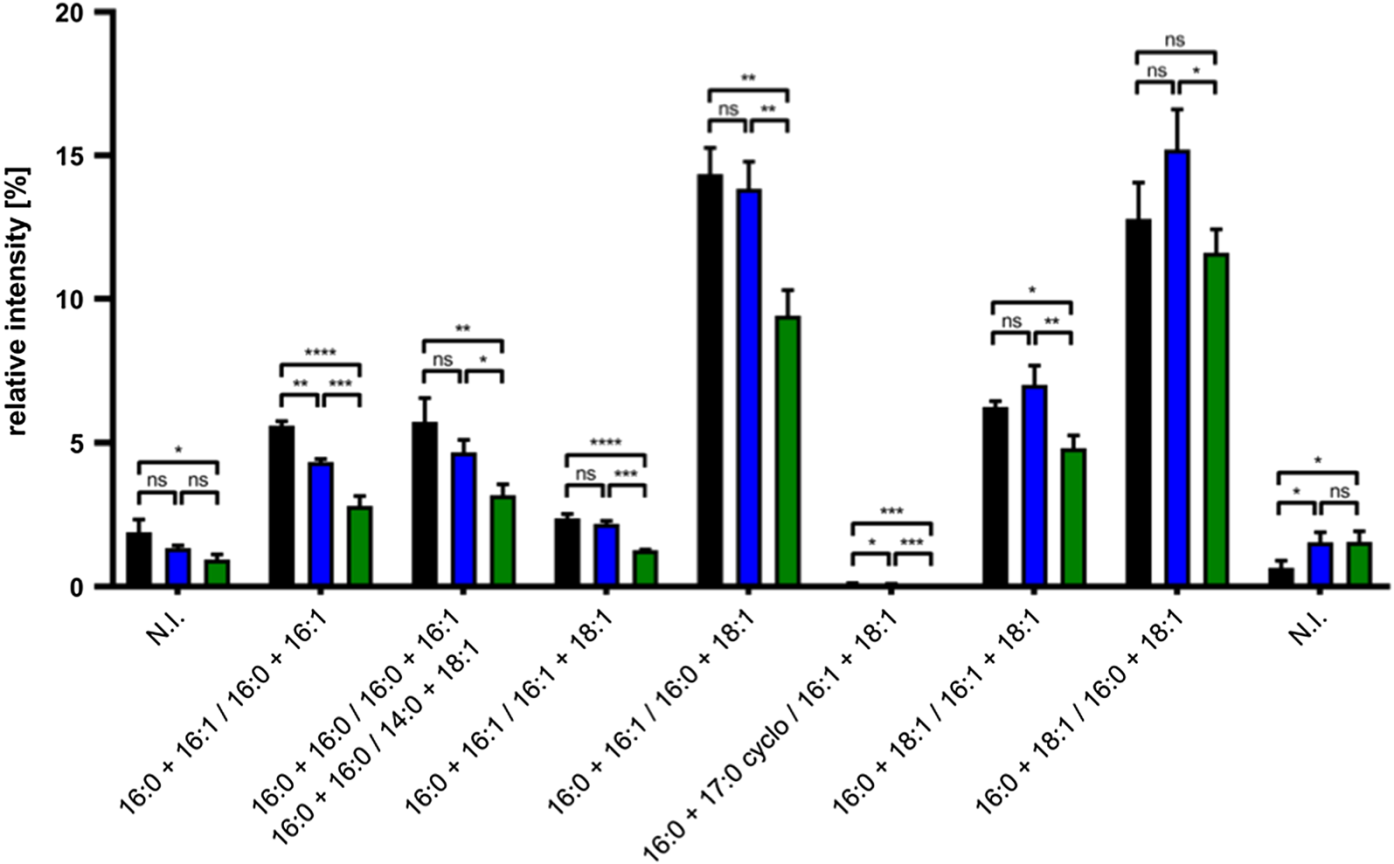
CL phospholipid FA analysis of C41(DE3) (black), C41(DE3)∆(*ompF*) (blue) and C41(DE3)∆(*ompF*-*acrAB*) (green). Data was analyzed using a 2-way ANOVA followed by Dunnett’s multiple comparisons test. ns: not significant (p > 0.05), *p ≤ 0.05, **p ≤ 0.01, ***p ≤ 0.001, ****p ≤ 0.0001. Error bars represent SD of three independent biological replicates

Cells overexpressing the ABC transporter HlyB were chosen because here the most profound changes in membrane solubilization behavior were observed. The quantification of total amounts of lipid and protein in membrane samples from C41(DE3), C41(DE3)*∆*(*ompF*), and C41(DE3)∆(*ompF*-*acrAB*) revealed significant changes in the lipid to protein ratio. The deletion of *ompF* resulted in a 1.2-fold decrease (p-value = 0.0088) in lipid-to-protein ratio and the additional deletion of *acrAB* in a 3.6-fold decline (p-value = < 0.0001) of total lipid-to protein ratio (see Additional file 1: Table S2). Phospholipid classes were unchanged between genotypes and consistent with published values (see Additional file 1: Table S2) [25]. To access the fatty acid (FA) composition, phospholipids were acid-digested and quantified by GC–MS. As expected, palmitic acid (16:0) is the most abundant FA in all samples. However its concentration was strongly reduced in C41(DE3)∆(*ompF*-*acrAB*) with a concomitant increase of lauric acid (12:0) (Fig. 7a). Speciation analyses of each phospholipid family (PE, PG, CL) confirmed the global FA analysis and revealed additional changes. Full statistical analysis of the identified species is available in Additional Phospholipid Analysis (see Additional file 2). For PE and PG, we observed a decrease in phospholipids with 16:0+16:1 in C41(DE3)∆(*ompF*) and C41(DE3)∆(*ompF*-*acrAB*) compared to C41(DE3) (Fig. 7b, c). Concentration of some phospholipids containing cyclopropanated FA (17:0 cyclo and 19:0 cyclo in PE and PG) are increased in C41(DE3)∆(*ompF*) and C41(DE3)∆(*ompF*-*acrAB*) (Fig. 7b, c). For CL species, C41(DE3) and C41(DE3)∆(*ompF*) phospholipids showed a similar FA pattern whereas most of the differences were observed in C41(DE3)∆(*ompF*-*acrAB*) (Fig. 8). Given the complexity and diversity of phospholipid species, we performed a principal component analysis (PCA) on the whole dataset of phospholipid speciation analysis (Fig. 7d). The hierarchical clustering visualized three distinct groups, corresponding to each individual strain. Globally, C41(DE3)∆(*ompF*) FA pattern is statistically different from C41(DE3); however, in C41(DE3)∆(*ompF*-*acrAB*) FA pattern is further differentiated compared to the two other strains (Fig. 7d). Taken together, the significant increase in cyclopropanated FA and in lauric acid (12:0) at the expense of palmitic acid (16:0) in mutant host C41(DE3)∆(*ompF*-*acrAB*) suggest a more fluid membrane.

### Mass spectrometric analysis of the proteomes

The proteomes of C41(DE3), C41(DE3)∆(*ompF*), and C41(DE3)∆(*ompF*-*acrAB*) were analyzed by mass spectrometry. The abundances of 1411 different gene products were compared between the three strains.

**Fig. 9:**
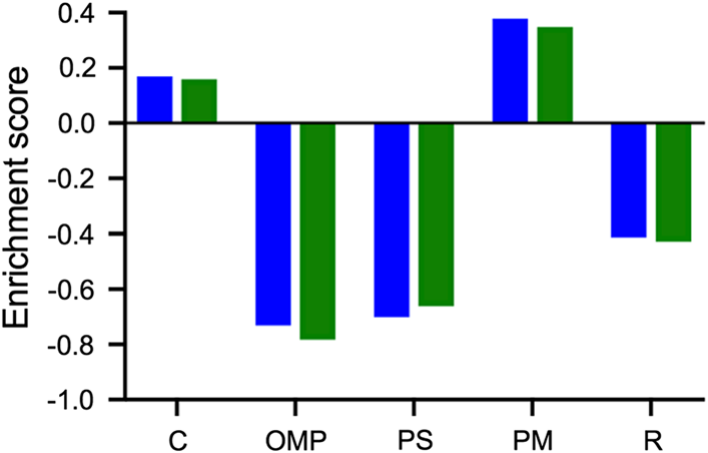
Summary of proteomics data. C41(DE3)∆(*ompF*) (blue) and C41(DE3)∆(*ompF*-*acrAB*) *(*green) compared to C41(DE3). Negative values mark a decrease, positive values an enrichment in protein in the corresponding cellular compartment. C: cytoplasm; OMP: outer membrane-bound periplasmic space; PS: periplasmic space; PM: plasma membrane; R: ribosome

The overall protein abundance patterns of C41(DE3)∆(*ompF*) and C41(DE3)∆(*ompF*-*acrAB*) were found to be similar, with no significant changes of single proteins except for AcrA and AcrB. However, large differences became apparent when the single and double deletion strains were compared to the parental strain C41(DE3) (Fig. 9). Beside the expected deletion of OmpF and additionally AcrA in C41(DE3)∆(*ompF*-*acrAB*), the two deletion strains showed lower abundances (21 proteins C41(DE3)∆(*ompF*) and 20 proteins in C41(DE3)∆(*ompF*-*acrAB*)) of the 22 proteins targeted to the periplasmic space (p-value = 1 E-6 for C41(DE3)∆(*ompF*) and 6.2 E-6 for C41(DE3)∆(*ompF*-*acrAB*)), and ribosomal protein (34 proteins, p-value = 0.00013 for C41(DE3)∆(*ompF*) and 35 proteins, p-value = 0.000088 in C41(DE3)∆(*ompF*-*acrAB*), but substantially higher abundances of 44 inner membrane associated proteins (35 proteins for C41(DE3)∆(*ompF*), p-value = 0.00029 and 31 proteins for C41(DE3)∆(*ompF*-*acrAB*), p-value = 0.00098). For example, the TMAO reductase TorT was found to be highly abundant in the parental strain samples, but could not be detected in the deletion strains (estimated fold change: > 300). Furthermore, periplasmic chaperones were significantly reduced, whereas members of the outer membrane protein insertion complex (BAM, BamB and BamD) showed an increased abundance in the deletion strains (BamB: 4.4 and 2.9 fold increased abundance in C41(DE3)∆(*ompF*) and C41(DE3)∆(*ompF*-*acrAB*) respectively; BamD: 2.3 and 2.2 fold increased abundance in the single and double deletion strain, respectively).

Some differences were also found in proteins involved in LPS and lipid biosynthesis and/ or transport. The abundance of the LPS-assembly protein LptD [26] (fold change C41(DE3)∆(*ompF*): 3.3, C41(DE3)∆(*ompF*-*acrAB*): 2.4) was increased, while that of the LPS export protein LptA [27], which mediates the transport of LPS to the outer membrane, was decreased (fold change C41(DE3)∆(*ompF*): 0.3, C41(DE3)∆(*ompF*-*acrAB*): 0.2). Moreover, the lysophospholipid lipase [28] (fold change C41(DE3)∆(*ompF*): 0.4, C41(DE3)∆(*ompF*-*acrAB*): 0.5) [29], was reduced.

In summary, the deletion of OmpF as well as AcrAB resulted in significant changes of the proteome, where the deletions resulted in a decrease of periplasmic and ribosomal proteins and an increase of membrane proteins.

## Discussion

By deleting both, OmpF and AcrB, in C41(DE3) host, we have not only removed two major contaminants but also improved the expression levels and purification yield of several membrane proteins. Surprisingly, deletion of OmpF in this strain revealed an improved growth to higher ODs, while the additional deletion of *acrAB* had no further effect (Fig. 1). The deletion of OmpF has been linked to an improved membrane integrity and tolerance towards certain substances and antibiotics by decreasing their influx [13, 30, 31], which provides a possible explanation for the improved growth of OmpF-depleted strains. In addition to the enhanced fitness of the cells and tolerance to MP production, we also observed a higher quality control and folding of the overproduced MP, a higher amount of incorporated target protein into the membrane (Figs. 2 and 5) and, consequently, a decrease in proteolytic degradation of the recombinant MP as exemplified in the case of SecYEG (Fig. 5). At biochemical level, we observed an improved solubilization efficiency with non-ionic detergents of HlyB, a protein mostly extracted with FC-14, a detergent unable to discriminate folded from non-folded MP [6, 32].

It is known that OmpF and AcrB are major contributors in membrane homeostasis. OmpF has been proposed to be part of the Mla lipid transport machinery [33] involved in membrane lipid asymmetry. Both, OmpF and AcrB, were also found to be involved in FA metabolism of *E. coli* by importing short chain FA into the periplasm from the extracellular space and by exporting FA to the extracellular medium, respectively [13, 14, 34]. Proteomic analysis revealed major changes in protein abundance in the periplasm and the inner membrane between C41(DE3) and the two mutant hosts. However, it failed to explain the different biochemical phenotypes observed between C41(DE3)∆(*ompF*) and C41(DE3)∆(*ompF*-*acrAB*). Therefore, and based on previous studies [35], we hypothesized that the phenotype of designed strains could be explained in part by changes in membrane organization. However, a simple phospholipid analysis based on head groups revealed no differences between strains. When phospholipids were digested and FA analyzed, differences were identified in C41(DE3)∆(*ompF*-*acrAB*), but not between C41(DE3) and C41(DE3)∆(*ompF*) in agreement with Tan et al. [13]. FA analysis for each phospholipid species, which is more precise than FA analysis after phospholipid acid-digestion, could eventually differentiate three distinct phenotypes (Fig. 3). For example, PG and PE are enriched in cyclopropanated FA in C41(DE3)∆(*ompF*) and C41(DE3)∆(*ompF*-*acrAB*) (Fig. 7b, c). Lipid bilayers containing cyclopropanated phospholipids have found to be more fluid, yet more ordered than their corresponding unsaturated precursors [36]. Consequently, *E. coli* membrane becomes more resistant to heat, acids, oxidants and osmotic shock [37]. Moreover, in C41(DE3)∆(*ompF*-*acrAB*), the membrane fluidity is further promoted by an increased concentration of lauric acid (12:0) at the expense of palmitic acid (16:0) containing phospholipids [38]. This enhanced membrane fluidity and stability may constitute an advantage to face MP overproduction. Indeed, the better MP insertion is reflected in a higher MP density, as demonstrated by sucrose density-gradient centrifugation (Fig. 8), while the lipid-to-protein ratio decreases. Consequently, solubilization and purification yields of recombinant MP are increased in C41(DE3)∆(*ompF*-*acrAB*).

To conclude, we present a new expression strain with enhanced membrane fluidity favoring MP membrane insertion and purification. While previous studies to improve overexpression of MP focused on transcriptional regulation [10, 11], we introduce instead an innovative approach based on the modulation of membrane composition. The expression strain constructed in this study may be useful for a large community of biochemists and structural biologists with potential applications in biotechnology.

## Materials and methods

### Construction of the expression strains C41(DE3)*∆*(*ompF*) and C41(DE3)*∆(ompF*-*acrAB)*

*OmpF*-deleted mutants were prepared from JW0192 *ΔompF* knockout (*E. coli* K12 BW25113) described in the Keio collection [39] by P1 transduction of C41(DE3) [40]. *OmpF* knockouts were selected using kanamycin resistance. Finally, kanamycin resistance was removed from *OmpF* knockouts by FLP-FRT recombination [41]. To additionally delete *acrAB* from C41(DE3)∆(*ompF*), the lambda-red recombinase system was employed, following published protocols [42]. Further details are provided in Additional file 1.

### Growth curves of *E. coli* strains

Growth curves were measured in 96-well plates on a microplate reader (Tecan) by monitoring the absorbance at 600 nm. Cell cultures were grown in 250 µL 2xYT-medium (10 g/L yeast extract, 16 g/L tryptone/peptone from pancreatic digestion, 5 g/L NaCl) at 37 °C and 650 rpm. Cultures were inoculated to OD_600_ of 0.15 and growth was monitored in triplicates over 10 h.

### Membrane protein overexpression

*E. coli* strains C41(DE3), C41(DE3)∆(*ompF*), and C41(DE3)∆(*ompF*-*acrAB*) were transformed with pBAD ABC transporter plasmids [17] and transformants were selected on agar plates containing 100 µg/mL ampicillin. Overnight cultures with 2xYT-medium (10 g/L yeast extract, 16 g/L tryptone/peptone from pancreatic digestion, 5 g/L NaCl) supplemented with 100 µg/mL ampicillin were inoculated with single colonies and incubated at 200 rpm, 37 °C for 15 h. Main cultures were grown in 5 L baffled flasks, containing 1 L of 2xYT-medium with 100 µg/mL ampicillin. Expression cultures were inoculated to OD_600_ of 0.1 and grown at 200 rpm, 37 °C until OD_600_ reached 2.5. The expression of the ABC transporters was induced by adding arabinose to a final concentration of 10 mM, incubation was continued for 3 h and cells were subsequently harvested by centrifugation. For overexpression of *E. coli* SecYEG translocon and YidC insertase, cells of the examined *E. coli* strains were transformed with the plasmids pEK20 (cysteine-less SecYEG) [43] or pEM183 (YidC) [44]. Further details are provided in Additional file 1.

### Isolation of membranes from *E. coli* cells

For all strains and overexpressed proteins, the same protocol for the extraction of the membrane-fraction was employed. Cells were resuspended in buffer P (50 mM NaH_2_PO_4_, pH 8, 300 mM NaCl) and lysed by passing through a cell-disruptor (Microfluidics) at 1.5 kbar. Membranes were harvested by a subsequent high-spin centrifugation step at 150,000×*g* for 90 min at 4 °C. Membrane pellets were homogenized in buffer P, supplemented with 10% glycerol, and stored at − 80 °C until further use. For SDS-PAGE, equal amounts (50 µg) of purified membranes were loaded on 10% gels and stained by Coomassie brilliant blue.

### Membrane protein purification

HlyB was purified as described in [24] with modifications that are summarized in Additional file 1. Overexpressed recombinant SecYEG and YidC proteins were purified as previously described [44, 45]. Further details are provided in Additional file 1.

### Membrane fractionation by density-gradient centrifugation

Continuous sucrose gradients (20–70% sucrose, 50 mM HEPES pH 7.4, 150 mM KCl, 5 mM MgCl_2_, and cOmplete protease inhibitor cocktail) were prepared in centrifuge tubes using the Gradient Station (BioComp Instruments). Membranes of the examined *E. coli* strains containing over-expressed YidC were loaded on top of the gradients and subjected to centrifugation for 16 h at 30,000 rpm (110,000×*g*) in SW40 rotor (Beckman Coulter) at 4 °C. The total gradient volume was 12 mL; of those 11 mL were fractionated from top to bottom using the Gradient Station (fraction volume 1 mL). The remaining volume (bottom) contained only non-separated and aggregated material and was excluded from the analysis. Proteins were precipitated by adding trichloracetic acid to the final concentration of 10%, pellets were washed with acetone, resuspended in SDS-PAGE loading buffer and incubated for 5 min at 95 °C prior to loading on a 15% SDS-PAGE gel and stained by Coomassie brilliant blue.

### Genome sequencing

Chromosomal DNA preparation and genome sequencing and analysis of *E. coli* C41(DE3)∆(*ompF*-*acrAB*) was performed as described elsewhere [10]. The genome sequence was deposited at NCBI (accession code SAMN11037806).

### Analysis of the proteomes of *E. coli* strains by quantitative mass spectrometric analysis

*E. coli* samples for mass spectrometry (four individual replicate cultures per group) were prepared as described in [46]. Further details are provided in Additional file 1. Data were deposited in the PRIDE database (accession PXD011437).

### Analysis of the lipidomes of *E. coli* strains by mass spectrometric analysis

#### Total lipid extraction

Lipids from HlyB overexpressing *E. coli* C41(DE3), C41(DE3)∆(*ompF*) or C41(DE3)∆(*ompF*-*acrAB*) (3 independent replicas of each sample were extracted using a procedure adapted from Bligh and Dyer [47]. Further details are summarized in SI.

#### Phospholipid separation, quantification and identification

Phospholipid separation by polar headgroup was performed on a Thermo Fisher Dionex UltiMate-3000 RSLC system. The separation of lipids was performed on a PVA-Sil column (150 × 2.1 mm I.D., 120 A) from YMC Europe GmbH thermostated at 35 °C. Chromatographic method was adapted from Ramos et al. [48]. For fatty acid quantification, phospholipids were digested and methylated using the *one pot* procedure described in [49]. Methylated fatty acids were analyzed in TraceGC Ultra coupled to an ITQ900 from Thermo Fisher equipped with an Agilent DB-5 capillary column. Further details are given in SI.

### Solubilization assays

HlyB was overexpressed in *E. coli* C41(DE3), C41(DE3)∆(*ompF*), or C41(DE3)∆(*ompF*-*acrAB*) and membranes were isolated as described above. Solubilization assays were performed in 96-well plates. The solubilization efficiency was assessed by measuring the optical density of the membrane solution. Data was normalized to the optical density after 100% solubilization in presence of 5% (w/v) SDS. Detergents were added to final concentrations ranging from 0 to 2% (w/v). Plates were incubated for 10 min and subsequently measured at 595 nm. Data was evaluated using GraphPad Prism 8 software (Graph Pad).

## Additional files

**Additional file 1.** The file contains detailed protocols for the lipidomics analyses, supplementary data for lipid species quantification, and detailed procedures supplementing the materials and methods section of the main manuscript.

**Additional file 2.** Relative quantifications of phospholipids (PE, PG, CL). The tables show the phospholipid assignations of the m/z peaks and the results of the statistical analyses.

## Data Availability

The genomics and proteomics datasets supporting the conclusions of this article are available in the NCBI database (SAMN11037806, https://www.ncbi.nlm.nih.gov/) and the PRIDE database (PXD011437, https://www.ebi.ac.uk/pride/archive/), respectively. The lipidomics datasets supporting the conclusions of this article are included in this article and its additional files.

## Abbreviations

DDM: dodecyl-β-maltoside
FA: fatty acid
FC: Fos-Choline
GC–MS: gas chromatography coupled to mass spectrometry
HlyB: hemolysin B
HlyBΔCLD: HlyB lacking the C39-like peptidase domain
IB: inclusion bodies
IMAC: immobilized metal ion affinity chromatography
MP: membrane protein
UMPS: unique membrane protein structure
PC: phosphatidylcholine
PE: phosphatidylethanolamine
CL: cardiolipin
