# Supplementary material for: Shaping the lipid composition of bacterial membranes for membrane protein production
Source: Microb Cell Fact. 2019 Aug 10;18:131. doi: 10.1186/s12934-019-1182-1 (PMC6689329; doi:10.1186/s12934-019-1182-1)
Supplement: Supplementary file 1 — Additional file 1. The file contains detailed protocols for the lipidomics analyses, supplementary data for lipid species quantification, and detailed procedures supplementing the materials and methods section of the main manuscript. [file 12934_2019_1182_MOESM1_ESM.docx]

**Additional file**

**Shaping the lipid composition of bacterial membranes for membrane protein production.**

Kerstin Kanonenberg^§1,2^, Jorge Royes^§3^, Alexej Kedrov^1^, Gereon Poschmann^4^, Federica Angius^3,5^, Audrey Solgadi^6^, Olivia Spitz^1^, Diana Kleinschrodt^1^, Kai Stühler^4^, Bruno Miroux^3*^, Lutz Schmitt^1*^

^1^ Institute of Biochemistry, Heinrich-Heine-University Duesseldorf, Germany

^2^ Current address: Université de Lyon, CNRS, UMR5086 “Molecular Microbiology and Structural Biochemistry”, IBCP, Lyon, France

^3^ Laboratoire de Biologie Physico-Chimique des Protéines Membranaires, UMR7099, CNRS, IBPC, Université Paris Diderot, Sorbonne Paris Cité, Paris, France

^4^ Molecular Proteomics Laboratory, Biologisch Medizinisches Forschungszentrum (BMFZ), Heinrich-Heine-University Duesseldorf, Duesseldorf, Germany

^5^ Current address: Department of Microbiology, Institute for Water and Wetland Research, Heyendaalseweg 135, 6525 Nijmegen, The Netherlands.

^6^ Université Paris-Saclay, Institut Paris Saclay d'Innovation Thérapeutique, INSERM, CNRS, - Plateforme SAMM - CHATENAY-MALABRY, France.

**Materials and Methods**

**Construction of the expression strains *E. coli* C41(DE3*)∆*(*ompF*) and *E. coli* C41(DE3*)∆*(*ompF-acrAB*)**

The plasmid pKD4 carrying a kanamycin resistance cassette was amplified using the forward and reverse overhang primers ACTTTTGACCATTGACCAATTTGAAATCGGACACTCGAGGTTTACATATGAGTAGGCTGGAGCTGCTTC and TTACGCGGCCTTAGTGATTACACGTTGTATCAATGATGATCGACAGTATGATGGGAATTAGCCATGGTCC, respectively. The genomic insertion of the kanamycin resistance gene using the pKD46-encoded lambda-red recombinase and its subsequent deletion with the pCP20-encoded Flp-recombinase were confirmed by PCR- and sequencing analysis using the primers CACATCGAGGATGTGTTG (forward) and GCCCTCTCGTTTGTTAG (reverse).

**Purification of HlyB from *E. coli* C41(DE3)∆(*ompF*-*acrAB*) membranes**

Membranes of 500 mL of cell culture were diluted to a final concentration of 10 mg/mL using buffer P. Membranes were solubilized with either 0.5 % (w/v) fos-choline 14, 1 % (w/v) DDM, 1 % (w/v) Triton X-100 or 1 % (w/v) LMNG for 1 hour at 8 °C. Non-solubilized material was removed by filtration (pore size: 0.45 µm) without the need of an additional centrifugation step. Solubilized membranes were diluted 1:4 with buffer P, supplemented with 2 mM imidazole and loaded on an IMAC (immobilized metal ion affinity chromatography) column (5 mL HiTrap Chelating HP, GE Healthcare, loaded with Zn^2+^). The column was washed with 8 mL of buffer P supplemented with 0.015 % (w/v) DDM or 0.003 % (w/v) LMNG and 2 mM imidazole. Non-specifically bound proteins were removed by washing with 18 mL of buffer P, supplemented with detergent and 40 mM imidazole. HlyB was eluted with buffer P containing the corresponding detergent and 25 mM EDTA.

**Overexpression of SecYEG and YidC**

Positive clones were selected on agar plates containing 100 µg/mL ampicillin. Overnight cultures with 2YT-medium and 100 µg/mL ampicillin were inoculated with single colonies and incubated at 200 rpm, 37 °C for 15 h. Main cultures were grown at 200 rpm, 37 °C in 5 L baffled flasks containing 1 L of 2YT-medium supplemented with 100 µg/mL ampicillin. Protein expression of SecYEG and YidC was induced by adding arabinose to a final concentration of 10 mM and IPTG to a final concentration of 1 mM, respectively. Cells were further incubated for 3 h and subsequently harvested by centrifugation.

**Purification of SecYEG and YidC from *E. coli* membranes**

Isolated membranes were solubilized with 1 % DDM in 50 mM HEPES pH 7.4, 300 mM KCl, 100 µM TCEP and complete protease inhibitor cocktail, and incubated with Ni^2+^-NTA agarose (Qiagen) in presence of 5 mM imidazole. After extensive wash with 50 mM HEPES pH 7.4, 150 mM KCl, 0.1 % DDM, and 20 mM imidazole, the imidazole concentration was increased to 300 mM to elute proteins of interest. Protein concentrations were determined using NanoDrop UV-Vis spectrophotometer based on calculated extinction coefficients (71,000 M^-1^ cm^-1^ for the SecYEG complex, and 96,000 M^-1^ cm^-1^ for YidC).

**Analysis of the proteomes of *E. coli* strains by quantitative mass spectrometric analysis**

Proteins from bacterial cells were extracted with lysis buffer (30 mM tris(hydroxymethyl)aminomethane, 2 M thiourea, 7 M urea, and 4 % (w/v) 3-[(3-cholamidopropyl)dimethylammonio]-1-propanesulfonate, pH 8.5). 5 µg of the proteins were loaded and briefly separated in a polyacrylamide gel (about 5 mm running distance). Bands from silver-stained gels were cut out, de-stained, washed, reduced, alkylated with iodoacetamide and proteins were digested with trypsin. Peptides were extracted from the gel and ~500 ng per sample analyzed by liquid chromatography coupled mass spectrometry (analogous to [1]). An Ultimate 3000 rapid separation liquid chromatography system (RSLC, Thermo Scientific) was used for sample separation on a 20 cm C18 chromatography column over a 2 h gradient. Peptides were directly injected into a QExactive plus (Thermo Scientific) triple quadrupole Orbitrap hybrid mass spectrometer via an electrospray nano source interface. The mass spectrometer was operated in data dependent positive mode. After acquisition of survey spectra at a resolution of 70,000, up to 10 precursors were isolated by the quadrupole, fragmented by higher-energy collisional dissociation and fragment spectra recorded at a resolution of 17,500 in the Orbitrap analyzer.

Database searches and quantification of precursor ion intensities was done within the MaxQuant environment (version 1.6.1.0, MPI for Biochemistry, Planegg, Germany) with standard parameters if not stated otherwise. The UniProt KB proteome dataset for *E. coli* BL21 (UP000002032, downloaded on 27th of June 2018), supplemented with an entry for HlyB was used for spectra identification. The ‘match between runs’ option was enabled as well as label-free quantification. Proteins and peptides were accepted at a false discovery rate of 1 %. For quantitative analysis only proteins were considered showing at least 2 different identified peptides and four valid values in at least one sample group. Statistical analysis (ANOVA) was performed within Perseus 1.6.2.2 (MPI for Biochemistry, Planegg, Germany) and the R environment (version 3.4.1, the R Foundation for Statistical Computing, post-hoc test) on log2 normalized intensities; missing values were filled in before with a downshifted (1.8 standard deviations) normal distribution (width 0.3 standard deviations). A one-dimensional annotation enrichment [2] was calculated within Perseus 1.6.2.2 on the basis of annotations provided by UniProt KB (downloaded on 8^th^ October, 2018). The mass spectrometry proteomics data have been deposited to the ProteomeXchange Consortium via the PRIDE [3] partner repository with the dataset identifier PXD011437. Coverage of the *E. coli* proteomes was approximately 40 %.

**Total lipid extraction**

Chloroform (1.25 mL) and methanol (2.50 mL) were sequentially added to a membrane suspension (1.00 mL) in phosphate buffer (50 mM, pH 8.0) containing 1 M NaCl. Samples were vortexed for 10 min at room temperature and chloroform (1.25 mL) and water (1.25 mL) were added. The organic phase (lower phase) was collected and the extraction procedure was repeated on the remaining aqueous phase. Combined organic layers were evaporated to dryness under an argon stream and stored at -20 °C under inert atmosphere.

**Analysis of the lipidomes of *E. coli* strains by mass spectrometric analysis**

Phospholipids for standard solution preparation, cardiolipin (CL) disodium salt from bovine heart, L-α-phosphatidylethanolamine (PE) and L-α-phosphatidylglycerol sodium (PG) both from chicken egg, were purchased from Avanti Polar Lipids. Stock solutions of each phospholipid were prepared in chloroform at 0.5, 0.4, 0.3, 0.2, 0.1, 0.05 and 0.025 mg/mL.

Organic HPLC grade solvents, *n*-heptane, chloroform and LC/MS grade methanol and absolute ethanol were purchased from VWR International. ULC-MS grade 2-propanol and water were purchased from Biosolve Chemicals. LC-MS grade acetic acid and triethylamine (99.5 % purity) were purchased from Sigma-Aldrich.

Flow rate was set to 0.4 mL/min and 5 µL of sample (diluted 1200-fold for PE quantification and 600x for PG and CL quantification) was injected. The mobile phase composition and the chromatographic program are summarized in Table 1. An isopropanol rinsing phase followed by a column equilibration phase were added at the end of the chromatographic program and performed before injecting a new sample.

Table S1: Mobile phase composition and chromatographic program.

| Time (min) | **A^a^** | **B^b^** | **C^c^** | **D^d^** |
| --- | --- | --- | --- | --- |
| 0 | 98 | 2 | 0 | 0 |
| 2 | 98 | 2 | 0 | 0 |
| 8 | 12 | 88 | 0 | 0 |
| 22 | 0 | 60 | 40 | 0 |
| 26 | 0 | 60 | 40 | 0 |
| 27 | 0 | 0 | 0 | 100 |
| 29 | 0 | 0 | 0 | 100 |
| 30 | 98 | 2 | 0 | 0 |
| 44 | 98 | 2 | 0 | 0 |

^a^ *n-*heptane/2*-*propanol (98:2 v/v)

^b^ chloroform/2-propanol (65:35 v/v)

^c^ methanol/water (95:5 v/v)

^d^ 2-propanol

All mobile phases except D contain 0.08% v/v triethylamine and 1.00% v/v acetic acid.

*Detection*

Separated phospholipids were analyzed with a dual detection system consisting of a Corona-CAD Ultra detector and LTQ-Orbitrap Velos Pro MS detector equipped with an H-ESI II probe from Thermo Fisher Scientific. After separation, HPLC effluent was split using a mixing tee, 250 µL/min entered to the Corona-CAD detector and 150 µL/min entered the mass spectrometer. To increase the flow entering the corona-CAD detector and maintain good aerosol stability, absolute ethanol was added with a second mixing tee with a flow of 200 µL/min.

The corona-CAD nebulizer was set to 30 °C and 5 bar of nitrogen pressure. The LTQ-Orbitrap Velos Pro MS detector spray voltage was set to 3.3 kV. Probe temperature was set to 200 °C. Sheath, auxiliary and sweep gas flow rates were set to 20, 8 and 0 (a.u.), respectively. Capillary temperature was set to 325 °C and S-lens RF level to 60 %. Analysis was performed in negative mode to obtain structural information on phospholipid fatty acid chains. The MS detector, equipped with two combined analyzers (a double linear ion trap, LTQ Velos Pro, and an orbital trap, Orbitrap^®^), provided fast fragmentation at low resolution and complete high-resolution fullscan spectra at the same time. Detection was carried out either in full MS Scan (100,000 resolution) and data dependent MS^2^ or MS^3^ with collision induced dissociation in the CID fragmentation (collision energy set to 35).

*Phospholipid identification and quantification*

The used chromatographic method separates the phospholipids by their polar headgroups. Retention time was calibrated using commercial PG, PE and CL standards. Subsequently, the fatty acid species were assigned an quantified for each family of phospholipids. Total phospholipid concentration was obtained and reported to total protein concentration on membrane extracts, determined by bicinchoninic acid assay (Table 2).

**Table S2**. Phospholipid identification and quantification in function of polar head. Results from three independent biological replicates are given as average ± standard deviation.

|  | PE  (% mol) | PG  (% mol) | CL  (% mol) | lipid-to-protein (w/w) |
| --- | --- | --- | --- | --- |
| C41(DE3) | 75.9±1.2 | 15.3±0.5 | 8.8±1.2 | 0.310±0.018 |
| C41(DE3)Δ(*ompF*) | 74.5±1.8 | 15.4±1.0 | 10.1±0.9 | 0.257±0.008 |
| C41(DE3)Δ(*ompF-AcrAB)* | 72.6±6.7 | 15.2±3.9 | 12.1±3.2 | 0.174±0.028 |

Phospholipid species identification was performed as previously described [4], combining high-resolution mass detection in full scan mode and MS^2^/MS^3^ fragmentation in data dependent mode. In minor *m/z* peaks, MS^n^ fragmentation was not always available. In these cases, only the number of carbons and unsaturations of the species were indicated. Sn1 and sn2 acyl chain position were not determined. Oxidised CL identification was performed as previously described [4, 5].

*Fatty acid quantification*

Fatty acid methyl esters (FAME) were prepared from phospholipid extracts using the method described by Ichimura *et al. [6].* The phospholipid extract (1 mL) was evaporated to dryness under an argon stream and re-solubilized in anhydrous toluene (0.2 mL). Methanol (1.5 mL) and a 8% w/w solution of chlorhydric acid in methanol (0.3 mL) were sequentially added. Samples were incubated at 45 °C for 14h. Then, water was added (1 mL) and fatty acid methyl esters were extracted with hexane (2x0.5 mL). The samples were evaporated to dryness under an argon stream, stocked under inert atmosphere at -20°C and re-solubilized in hexane (1 mL) prior to GC-MS analysis.

FAME were separated using a TraceGC Ultra coupled to an ITQ900 from Thermo Fisher equipped with an Agilent DB-5HT capillary column (30m x 0.25 mm ID, 0.25 µm film thickness). The samples (1µL) were injected in splitless mode. The temperature of the injector and the transfer line was kept at 250 °C. Helium was used as carrier gas (1 mL/min). The MS detector temperature was adjusted to 200 °C. The GC temperature program is shown in detail in Table 3.

Table S3: GC temperature program.

|  | Rate (°C/min) | Temperature (°C) | Hold time (min) |
| --- | --- | --- | --- |
| Initial | - | 85 | 4.0 |
| Ramp 1 | 9.0 | 150 | 0.0 |
| Ramp 2 | 3.0 | 210 | 0.0 |
| Ramp 3 | 9.0 | 280 | 0.0 |

All FA present were identified by their characteristic retention time (t_R_) and compared to a commercial Bacterial Acid Methyl Esters (BAME) standard mixture from Matreya LCC. The 18:1 peak was compared to a *cis-*vaccenic commercial standard obtained in Matreya LCC. FAME and *cis-*vaccenic calibration curves were built using seven points, containing from 3 to 400 µg/mL of each standard. Two different dilutions per sample were made (1/20 and 1/50) to allow the quantification of major and minor FA, respectively. Samples were prepared in triplicates using independent cell cultures.

**References**

1. Grube L, Dellen R, Kruse F, Schwender H, Stuhler K, Poschmann G: **Mining the Secretome of C2C12 Muscle Cells: Data Dependent Experimental Approach To Analyze Protein Secretion Using Label-Free Quantification and Peptide Based Analysis.** *J Proteome Res* 2018, **17:**879-890.

2. Cox J, Mann M: **1D and 2D annotation enrichment: a statistical method integrating quantitative proteomics with complementary high-throughput data.** *BMC Bioinformatics* 2012, **13 Suppl 16:**S12.

3. Vizcaino JA, Csordas A, Del-Toro N, Dianes JA, Griss J, Lavidas I, Mayer G, Perez-Riverol Y, Reisinger F, Ternent T, et al: **2016 update of the PRIDE database and its related tools.** *Nucleic Acids Res* 2016, **44:**11033.

4. Pulfer M, Murphy RC: **Electrospray mass spectrometry of phospholipids.** *Mass Spectrom Rev* 2003, **22:**332-364.

5. Tyurin VA, Tyurina YY, Jung MY, Tungekar MA, Wasserloos KJ, Bayir H, Greenberger JS, Kochanek PM, Shvedova AA, Pitt B, Kagan VE: **Mass-spectrometric analysis of hydroperoxy- and hydroxy-derivatives of cardiolipin and phosphatidylserine in cells and tissues induced by pro-apoptotic and pro-inflammatory stimuli.** *J Chromatogr B Analyt Technol Biomed Life Sci* 2009, **877:**2863-2872.

6. Ichihara K, Fukubayashi Y: **Preparation of fatty acid methyl esters for gas-liquid chromatography.** *J Lipid Res* 2010, **51:**635-640.
